# Activation of the *miR-371/372/373* miRNA Cluster Enhances Oncogenicity and Drug Resistance in Oral Carcinoma Cells

**DOI:** 10.3390/ijms21249442

**Published:** 2020-12-11

**Authors:** Shu-Chun Lin, Hsiao-Li Wu, Li-Yin Yeh, Cheng-Chieh Yang, Shou-Yen Kao, Kuo-Wei Chang

**Affiliations:** 1 Institute of Oral Biology, School of Dentistry, National Yang-Ming University, Taipei 11221, Taiwan; sclin@ym.edu.tw (S.-C.L.); lydia1229@gm.ym.edu.tw (H.-L.W.); liyin.yeh@gmail.com (L.-Y.Y.); ccyang@ym.edu.tw (C.-C.Y.); sykao@vghtpe.gov.tw (S.-Y.K.); 2 Department of Dentistry, School of Dentistry, National Yang-Ming University, Taipei 11221, Taiwan; 3 Department of Stomatology, Taipei Veterans General Hospital, Taipei 11217, Taiwan

**Keywords:** apoptosis, cancer, CRISPR, gene cluster, *miR-371/372/373*, promoter

## Abstract

Oral squamous cell carcinoma (OSCC) is among the leading causes of cancer-associated deaths worldwide. Family members in *miR-371/372/373* miRNA cluster, which is localized at human chromosome 19q13.4, are co-expressed in both human stem cells and malignancies. The individual miRNA in this cluster are also involved in modulating the pathogenesis of malignancies as either oncogenes or suppressors. The 19q13 region is frequently gained in head and neck cancers. High expression of *miR-372* and *miR-373* are survival predictors for OSCC. However, the role of the *miR-371/372/373* cluster in oral carcinogenesis remains to be fully investigated. We use the clustered, regularly interspaced, short palindromic repeats (CRISPR)-Cas9 system to establish OSCC cell subclones that had the *miR-371/372/373* cluster deleted. In addition, further subclones were established that had the promoter of this cluster deleted. Concordant silencing in SAS cells of *miR-371/372/373* decreased oncogenic potential, increased cisplatin sensitivity, activated p53, and upregulated the expression of Bad and DKK1. We also employed the CRISPR/dCas9 synergistic activation mediator system, which allowed robust transcriptional activation of the whole *miR-371/372/373* cistron. Upregulation of endogenous *miR-371/372/372* expression increased both oncogenicity and drug resistance. These were accompanied by a slight activation of AKT, β-catenin, and Src. This study identifies the oncogenic role of the *miR-371/372/373* cluster in OSCC. Using CRISPR based strategy can be a powerful paradigm that will provide mechanistic insights into miRNA cluster functionality, which will also likely help the development of targeting options for malignancies.

## 1. Introduction

Oral squamous cell carcinoma (OSCC) is one of the prevalent head and neck cancers worldwide [1,2]. miRNAs are non-coding RNAs that play various important roles in regulating specifically-targeted transcription events during pathogenesis [1,2,3,4]. Overrepresentations of chromosomes 19q is a common event during head and neck carcinogenesis that includes nodal involvement [5]. Multiple members of the chromosome 19q13 miRNA cluster (C19MC), which consists of more than 40 miRNAs, have been identified as being involved in modulating a wide variety of biological activities; these include self-renewal, apoptosis, and oncogenesis [6,7,8]. *miR-371*, *miR-372*, and *miR-373*, all to be found in the *miR-371/372/373* cluster, which is around 1 Kbp in length, is close to the telomere of chromosome 19. Furthermore, C19MC is involved in modulating stemness, and is associated with the pathogenesis of a range of diseases and neoplasms [9,10]. In addition, a conserved murine homolog of the human *miR-371/372/373* cluster, which has been designated the *miR-290-295* cluster, is present in mouse genome [11].

Wingless-related integration site (Wnt)/β-catenin pathway is a pivotal cascade modulating differentiation and oncogenesis. Upon the binding of Wnt to receptors, the de-phosphorylated cytosolic β-catenin translocates into nucleus as active β-catenin, which transactivates T-cell factor/lymphoid enhancer factor (Tcf/Lef) elements to turn on the expression of Wnt targets [10]. Wnt signaling has been shown to direct upregulation of *miR-371/372/373* cluster members by transactivating Tcf/Lef elements in the promoter. Moreover, a positive regulation loop has been noted between Wnt and *miR-372*/*miR-373* in tumor cells [10]. The oncogenic roles of *miR-372*/*373*, which involves targeting of LATS2, have been well studies in various malignancies [12,13]. In OSCC, *miR-371/372/373* has been found to be up-regulated in tumors relative to control mucosa [2,14,15,16,17]. In addition, *miR-372* and *miR-373* are known to be associated in OSCC with nodal metastasis, lymphovascular permeation, and a worse prognosis [15]. Moreover, the level of *miR-372* in body fluids has been validated as an OSCC biomarker that is related to the severity of tumors [14,16,18]. Upregulation of the *miR-371/372/373* cluster is also found in esophageal SCC [19]. A comprehensive survey has revealed that expression of the *miR-371/372/373* cluster residents is able to act as a biomarker for diagnosis and prognosis [20].

The members of the *miR-371/372/373* cluster have been shown to target a number of important genes such as LATS, p62, and ZBTB1A, which then drive oncogenic progression in OSCC [15,16,17]. Dickkopf 1 (DKK1) is a Wnt antagonist being known to suppress Wnt-associated pathogenesis [10,21,22,23,24]. The targeting of *miR-373* on DKK1 to enrich the neoplastic process has been shown in tongue carcinoma [22]. However, a divergence between DKK1’s functions and DKK1’s expression profile is also present in OSCC [21,22,23,24]. *miR-371/372/373* members are co-upregulated in OSCC, but the various effects of *miR-371/372/373* cistron during the regulation of target genes still remain elusive.

A number of lines of evidence seem to indicate that the *miR-371/372/373* cluster, as a whole or as one or more of its members, plays complicated or perhaps biphasic roles in tumorigenesis. Downregulation of *miR-371/372/373* promotes self-renewal capacity and metastatic colonization by colorectal carcinoma cells [25]. *miR-372* has also been shown to target a variety of oncogenes and thus can be involved in the suppression of various cancers [26,27,28]. Therefore, an investigation of the impact of the *miR-371/372/373* cluster and its promoter on OSCC cells is required to provide valuable insights into OSCC oncogenesis.

The CRISPR (clustered, regularly interspaced, short palindromic repeats)/Cas9 system uses Cas9 and a small guide RNA (sgRNA) to form a complex that recognizes a complimentary DNA sequence; this complex then cuts the DNA double strain to initiate a repair process that brings about gene deletion [29]. Furthermore, deactivated Cas9 (dCas9), which retains the protein’s DNA binding affinity, has been developed into the CRISPR-dCas9 SAM (synergistic activation mediator) system that allows gene activation [30]. Upon the binding of the sgRNA to a target in a transcription start region, the MS2 binding ring in backbone recruits the activation factor complex that can bring about promoter activation [31]. CRISPR systems have been widely used in transgenic technologies, gene editing, high throughput screening, the identification of new biomarkers, and a variety of translational clinical applications [32,33]. The concordant silencing of the members of the *miR-371/372/373* cluster by deletion of either the whole cistron or the promoter using a CRISPR/Cas9 editing strategy was carried out in this study [3,17]. *miR-371/372/373* silencing was found to decrease OSCC oncogenicity and cisplatin sensitivity. On the contrary, *miR-371/372/373* upregulation, when brought about by the CRISPR-dCas9 SAM system, results in the opposite effect.

## 2. Results

### 2.1. Establishment of the Gene Deletion Subclones

**Figure 1:**
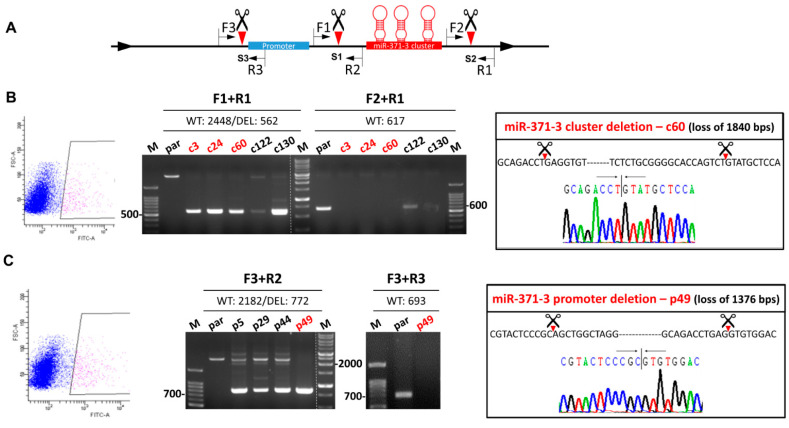
Deletion of the *miR-371/372/373* cluster and the *miR-371/372/373* promoter in SAS cell using clustered, regularly interspaced, short palindromic repeats (CRISPR)/Cas9 system. (**A**) Schematic diagram of the approach used. Green box, promoter; Red box, *miR-371/372/373* cluster. Scissors and triangles indicate the predicted S1–S3 cleavage sites. F, location of the forward primers; R, location of the reverse primers. (**B**,**C**) Deletion of the *miR-371/372/373* cluster and the *miR-371/372/373* promoter, respectively. Lt, sorting of GFP^+^ cells being blocked. Middle, electrophoretic gel illustrating the amplicons generated by the different combinations of primers. Red color labels the certified homozygous deletion after repeat examination. Rt, sequencing results. The truncations of the gene segments in the c60 and p49 subclones are shown.

The strategy used for deleting the gene cluster and the promoter by means of CRISPR/Cas9 system is presented in Figure 1A. Cleavage of Cas9 at S1 and S2 should result in cluster truncation. Similarly, cleavage at S1 and S3 sites should result in promoter truncation. SAS OSCC cells exhibit green fluorescence were sorted (Figure 1B,C, Lt). The sorted cells were then re-suspended to give single cell aliquots and these were grown to confluence. The successful deletion of each gene segment in a stable subclone was detected by PCR analysis using defined primers. Using the DNA of c3, c24, c60, and c130 subclones, amplification using the F1/R1 primer pair generated amplicons that were ~1.9 Kbs smaller than that of parental cells, while in parallel the F2/R1 primer pair did not yield an amplicon. This shows that there had been successful deletion of the gene cluster in these subclones (Figure 1B, Middle). Sequencing of the c60 subclone confirmed the loss of 1840 bps of DNA that includes the *miR-371/372/373* cluster (Figure 1B, Rt). Using the DNA of p49 subclone, amplification using the F3/R2 primer pair generated an amplicon that was ~1.4 Kbs smaller than that of the parental cells, while in parallel the F3/R3 primer pair did not yield an amplicon (Figure 1C, Middle). Sequencing of this amplicons confirmed the loss of 1376 bps of DNA that includes the *miR-371/372/373* cluster promoter region (Figure 1C, Rt). Other examples of the p series subclones, including p5, p29, and p44, were found to have heterozygous deletions and identifying promotor deletion subclones by cell sorting was unsuccessful with these clones (not shown).

### 2.2. Deletion Attenuates Oncogenicity and Upregulates DKK1

**Figure 2:**
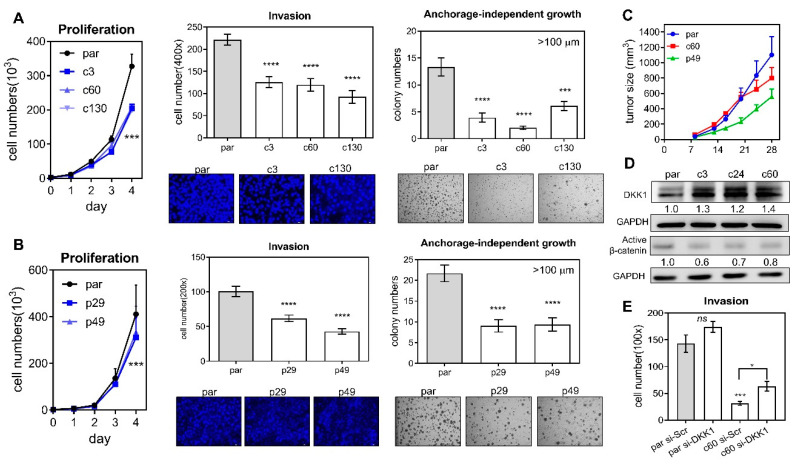
Phenotypic analysis of the deleted subclones. (**A**,**B**) Deletion of the *miR-371/372/373* cluster and the *miR-371/372/373* promoter, respectively. Lt, proliferation; Middle, invasion; Rt, anchorage-independent growth. Upper, quantification; Lower, representative fields. Invasion, ×100; anchorage-independent growth, ×50. (**C**) Subcutaneous xenografts in nude mice. Four or eight tumors in each group. (**D**) Western blot analysis of DKK1 and β-catenin expression in parental cells and the cluster deleted subclones. (**E**) Invasion assay of parental cells and the c60 subclone after treatment of 60 mM si-Scr or si-DKK1 oligonucleotides for 48 h. par, parental cell. *, *p* < 0.05; ***, *p* < 0.001; ****, *p* < 0.0001.

An analysis of growth, invasion, and anchorage-independent growth was performed on the parental cells and the various deletion subclones. Deletion of the *miR-371/372/373* cluster (Figure 2A), as well as deletion of its promoter (Figure 2B), was able to significantly attenuate proliferation (Figure 2A,B Lt), invasion through a matrigel barrier (Figure 2A,B Middle) and the ability for growth in an anchorage-independent culture environment (Figure 2A,B Rt). Furthermore, the xenografic growth potential of c60 and p49 subclones was found to be lower than that of their parental cells (Figure 2D). Thus, the *miR-371/372/373* cluster and its promoter are able to modulate oncogenesis in OSCC cells. Analysis of the potential targets of *miR-371/372/373* showed an unequivocal upregulation of DKK1 in deleted subclones (Figure 2D). In addition, active β-catenin was found to be decreased in the same subclones. However, the changes in expression of LAST2, SPOP, YOD1, ZBTB7A, and p62 proteins were found to be quite diverse in subclones (Figure S1). The mRNA expression of the *MYADM* and *PRKCG* genes, which are localized near to and downstream of the *miR-371/372/373* cluster, was not consistently affected by homozygous or heterozygous deletion of the promoter (Figure S2). The decreased invasiveness of the c60 subclone was able to be rescued by knockdown of DKK1 (Figure 2E), which suggests that there is an association between the *miR-371/372/373*-DKK1 cascade and the aggressiveness of OSCC cells.

### 2.3. Deletion Increases Cisplatin-Induced Cell Apoptosis

**Figure 3:**
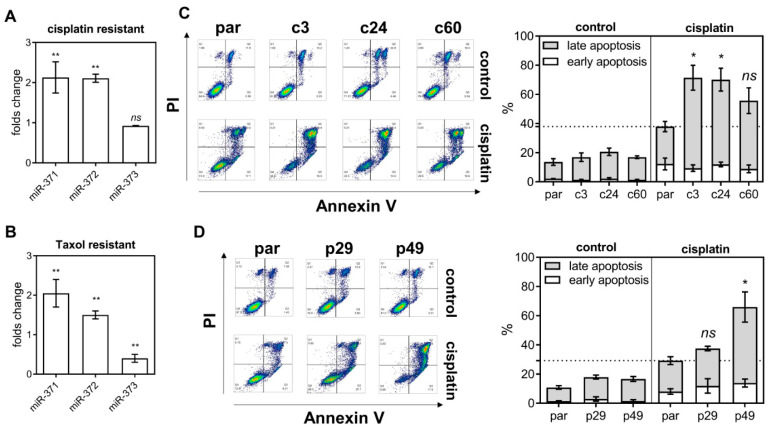
*miR-371/372/373* deletion and cisplatin treatment induce apoptosis. (**A**,**B**) The expression of the individual members of the *miR-371/372/373* cluster in the cisplatin resistant SAS cell subclone and the taxol resistant SAS cell subclones, respectively. (**C**,**D**) Flow cytometry analysis of the apoptopic cell fraction in the *miR-371/372/373* cluster deletion and the *miR-371/372/373* promoter deletion subclones, respectively. Lt, representative sorting diagrams; Rt, quantitation. *, *p* < 0.05; **, *p* < 0.01.

In cisplatin resistant and taxol resistant SAS subclones, the levels of *miR-371* and *miR-372* expression are higher than in the parental cell (Figure 3A,B, respectively). Early apoptosis and late apoptosis populations were studied by flow cytometry using sorting for Annexin V/PI labeling cells (Figure 3C,D, Lt). Both the c-subclones and p-subclones exhibited slightly higher late apoptosis populations relative to the parental cells (Figure 3C,D, Rt). On treatment with 15 μM cisplatin for 48 h, the late apoptosis population increased drastically in deleted subclones relative to parental cells. It should be noted that, in the p29 heterozygous deletion subclone, there was no significant increase in cisplatin induced apoptosis (Figure 3D). On treatment with AG1478, the apoptotic population in deletion subclones was higher than in the parental cells (Figure S3).

### 2.4. p53 Activation and Bad Upregulation Underlie Deletion Associated Apoptosis

**Figure 4:**
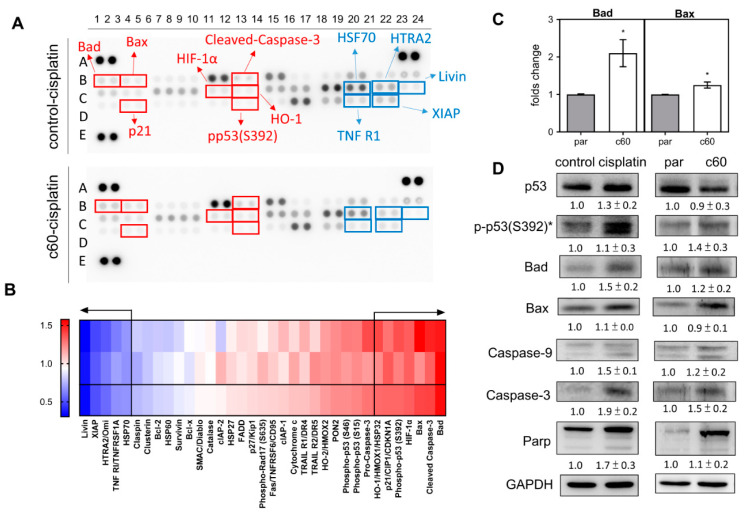
Analysis of various apoptosis associated proteins. (**A**) The Apoptosis Protein Array. Red rectangles mark the upregulated proteins, while the blue rectangles mark the downregulated proteins. (**B**) Heatmap of the protein changes in the c60 subclone relative to parental cells following cisplatin treatment. At the bottom are the average of the spot duplicates presented above. Proteins showing a change in abundance that exceeds 30% are separated from the others by arrows. Lt, gradient bar. (**C**) qPCR analysis of *Bad* and *Bax* mRNA expression. (**D**) Western blot analysis of p53, pp53 (s392), Bad, Bax, Caspase-9, Caspase-3, and Parp expression. Comparisons are made between the control cells and the cisplatin treated cells, and between the parental cells and the c60 subclone cells. Data shown are means ± SE with at least triplicate analysis. *, normalized against the p53 level. par, parental cells.

The cell lysates isolated from the control cells and the c60 subclone were subjected to array analysis to identify factors that could be associated with apoptotic induction (Figure 4A). Among the 35 proteins analyzed, there were seven upregulated proteins, namely Bad, Bax, HIF-1α, HO1, p21, pp53(S392), and cleaved-caspase 3. Furthermore, there were also five downregulated proteins, namely HSF70, HTRA2, Livin, TNFR1, and XIAP. Both groups had changes of expression of >30% (Figure 4B). qPCR analysis indicated that there was a significant upregulation of *Bad* and *Bax* mRNA expression in the c60 subclone relative to the parental cells (Figure 4C). Western blot analysis showed that there were increased protein levels of pp53(S392), Bad, Caspase-9, Caspase-3, and Parp, both in the cisplatin treated parental cells and in the c60 subclone. Therefore, it seems that p53 activation and Bad upregulation are likely to underlie the *miR-371/372/373* deletion apoptosis and the cisplatin induced apoptosis.

### 2.5. Endogenous miR-371/372/373 Expression Increases Oncogenicity, Drug Resistance and Signal Activation

**Figure 5:**
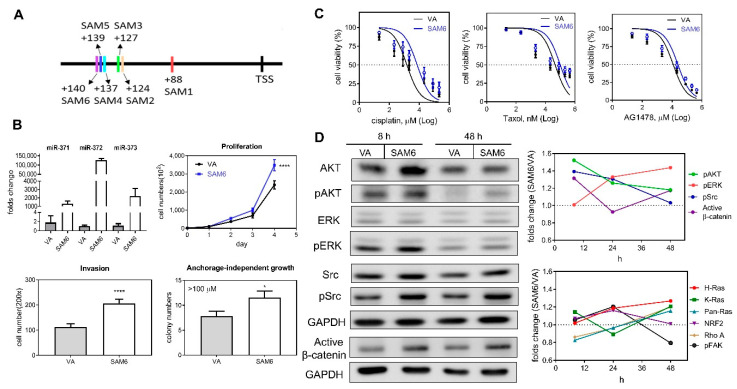
Transactivation of the *miR-371/372/373* promoter in SAS cells using the CRIPR/dCas9 synergistic activation mediator (SAM) system. (**A**) Schematic diagram illustrating the strategy used. The six oligonucleotide targeting sites for SAM functioning are marked as SAM1–SAM6. (**B**–**D**) Comparisons across cells transfected with the SAM6 construct and vector alone (VA). (**B**) Lt Upper, Expression of *miR-371/372/373* members. Others, assays measuring proliferation, invasion, and anchorage-independent growth. *, *p* < 0.05; ****, *p* < 0.0001. (**C**) Dose-response plots for cisplatin, taxol, and AG1478. Cells viability was measured by the MTT assay. Doses of drugs are Log_10_ transformed. (**D**) Western blot analysis. Lt, A representative analysis illustrating the changes in AKT, pAKT, ERK, pERK, Src, pSrc, and active-β-catenin expression at 8 h and 48 h following transfection. Rt, quantification of the signals over the time course from using at least duplicate analysis. Upper, pAKT, pERK, pSrc, and active-β-catenin. Lower, Ras families, NRF2, Rho A, and pFAK. Data shown are mean values from at least duplicate analysis. Representative analysis is described in Figure 5S.

A SAM activation strategy was adopted to increase endogenous *miR-371/372/373* expression by promoter transactivation. Six potential sites localized in 5′- region of the transcription start site (TSS) were used for SAM activity and are illustrated in Figure 5A. Plasmids containing different guide oligonucleotides and SAM components were generated and these were designated SAM1 to SAM6 (Table S3). After transfection, qPCR analysis revealed a drastic upregulation of *miR-371/372/373* expression following induction of SAM2–SAM6 (Figure S4). Upregulation of *miR-372* was greater than the upregulation of either *miR-371* or *miR-373*. The upregulation of *miR-371/372/373* expression following transfection with SAM4–SAM6 for 24 h was particularly obvious (Figure 5B, Lt Upper). The proliferation, invasion, and anchorage-independent growth of the cells was increased after the transfection with SAM6 (Figure 5B). In addition, cisplatin, taxol, and AG1478 resistance were increased following transfection of SAM6 into these cells (Figure 5C). Western blot analysis showed a limited increase in the protein levels of AKT, Src, and β-catenin 8 h after transfection to induce activation (Figure 5D). During the time courses, the activation of Ras, NRF2, and FAK was not detectable (Figure 5D, Rt Lower; representative analysis in Figure S5).

## 3. Discussion

Although the functions and expression of the members of the *miR-371/372/373* cluster are controversial in different types of malignancies [13,21,26,27,34], this study, by using a CRISPR editing strategy to delete the *miR-371/372/373* cluster or *miR-371/372/373* promoter, has unequivocally been able to identify a suppression of oncogenicity that followed deletion of these chromosomal regions. In line with these findings, information derived from using the SAM activation system also substantiates the oncogenic roles of this miRNA cluster. Apart from the C19MC cluster, it is known that other miRNA clusters, such as the *miR-17-92* and *miR-134-miR-655* clusters, also harbor members that are important for cancer pathogenesis [1,3,35,36,37]. Thus, it is clear that the using of CRISPR knockout/activation strategies would be helpful when elucidating the overall functions of these miRNAs and their targets.

LATS2, YOD1, ZBTB7A, and p62 have been reported in various studies to be targets of *miR-371/372/373* cluster members [13,16,17,34], but the changes in expression found after miRNA depletion are not remarkable. SPOP and ZBTB7A have been identified by multiple prediction algorithms as having the highest scores for *miR-371/372/373* targeting (detailed analysis not shown), but the level of SPOP expression was not altered following *miR-371/372/373* deletion. The *miR-372*-ZBTB7A-Trail-R2 axis has been shown to be a critical apoptosis pathway in our previous study [17]. The ZBTB7A is not modulated by the *miR-371/372/373* cluster; in fact, the screening and validation studies specifically identify the p53-Bad-Caspases axis as potential cascade underlying the induction of apoptosis in the *miR-371/372/373* deficient subclones. Previous efforts have mainly addressed the impact of individual miRNAs within a cluster on oncogenic phenotypes [16,17,21,25,26,27,28,34]. However, this work shows the coordinated net effects of all of the miRNAs within the same cistron that are driven by the same promoter. Although the concordant expression of *miR-371/372/373* exerts oncogenic stimuli to cells, the molecular mechanisms that are involved could deviate from those modulated by a solitary miRNA. Competition among the same miRNA family could underlie any such discrepancies. This study only provides evidence derived from various cell subclones obtained from the SAS cell line. Clonal expansion potential in other OSCC cells would seem to be abrogated after the deletion of a positive regulator (*miR-371/372/373*) and this may hinder the establishment of stable cell subclones. Alternatively, the wild type p53 function that exists in the SAS cell may ensure the occurrence of DNA repair after nuclease cleavage, which would benefit the survival of cells by protecting against the attack by Cas9.

Clues identified in this study indicate DKK1 to be one of the targets of *miR-371/372/373* [10,22]. The β-catenin inactivation consequential to DKK1 activation can also be seen. Furthermore, the cell invasion capability associated with *miR-371/372/373* deletion is able to be rescued by the knockdown of DKK1. Furthermore, a previous study has identified the suppressor role of DKK1 in OSCC [21], and this is in agreement with the present findings. DKK1 upregulation has also been reported to be an independent predictor of a poor OSCC prognosis based on the TCGA dataset [23]. Although prediction algorithms depict that each member of *miR-371/372/373* cistron targets the same sequences in the 3′ un-translation region of DKK1 gene, a direct evidence specifying DKK1 to be one of the targets of *miR-371/372/373* cluster should be provided in future studies [10,16,17,22]. Studies have also highlighted a positive regulation loop between β-catenin and the *miR-371/372/373* cluster [10]. In this context, the role of DKK-Wnt in the neoplastic process remains controversial and therefore a comprehensive molecular approach linked to tissue analysis is required to address what seems to be the versatile interplay within the DKK1-β-catenin-*miR-371/372/373* loop across different tumor microenvironments.

Studies have identified individual members of the *miR-371/372/373* cluster as potential tumor suppressors [25,26,27,28], and it would seem to be a good idea to use the CRISPR editing system to determine precisely the roles of the various members of the *miR-371/372/373* cistron in a range of tumors. It has been reported in lung carcinomas that silenced *miR-373* induced by DNA methylation in promoter contributes to cisplatin sensitivity and tumor growth suppression [38]. Epigenetic silencing of *miR-373* promoter resulting in the increased cell proliferation has also been shown in colorectal carcinoma [39]. Despite that the mechanisms regulating the methylation of CpG islands in promoters are important issues to be addressed, the de-methylation reagents used in experiments may contemporarily activate a wide panel of promoters to elicits non-specific reactivities [38,39]. Therefore, the CRISPR/Cas9 deletion system we adopted in this study to specifically silence the promoter activity in combination with the CRISPR/dCas9 SAM system to bring about the endogenous promoter activation would facilitate the functional elucidation of *miR-371/372/373* promoter. Both systems should also be used to analyze additional cell lines to validate the significances of *miR-371/372/373* promoter in oncogenicity and drug resistance of OSCC.

Under the same endogenous modulation of *miR-371/372/373* expression mediated by CRISPR/dCas9 SAM activation, the transcription level of *miR-372* seems to far surpass the level of the other two members. The presence of feedback regulation also has a confounding effect and one possibility is that the structural complexity of the pri-miRNA, the processing efficiency of the pri-miRNA, and the stability of the mature miRNA could all be involved in the differences in the abundance of each miRNA. Although the SAM induction is significant, it is clear that, under SAM control, oncogenicity does increase significantly, and at the same time, various oncogenic signals including β-catenin, only activated to a limited degree. Future work needs to explore the homeostasis amongst these signals and this will require determining ideal time courses for these assessments. The CRISPR/Cas9 editing system functions in the nucleus and this create difficulties in terms of genetic engineering and accessibility. Establishing stable cell subclone also remains somewhat challenging. There seem to have been many heterogeneities across the various cell subclones. While the SAM system induces endogenous expression through transient transfection that by passes the exogenous interference, its applications remain limited to promoter activation at present [30,31,33]. Newly developed CRISPR-based RNA-targeting may help by providing more effective editing of the mRNA transcript when it is used for diagnostic or prognostic applications [40]. This study annotates the oncogenic role of the *miR-371/372/373* cluster in OSCC cells using CRISPR-based approaches. DKK1-β-catenin and p53-Bad-Caspases were found to play a pivotal role in the downstream cascade of this oncogenic cluster.

## 4. Materials and Methods

### 4.1. Cell Culture, Reagents, and Phenotypic Assays

The SAS OSCC cell line was cultured as previously described [1,2]. Cisplatin and taxol were purchased from Sigma-Aldrich (St Louise, MO, USA). AG1478 EGFR inhibitor was purchased from Abcam (Cambridge, MA, USA). The cisplatin resistant and taxol resistant SAS subclones were those that we had previously established [17]. Analysis of cell growth, invasion, and anchorage-independent growth ability followed previously published protocols [17]. si-DKK1 (Cat No.: sc-37082) and si-Scr (Cat No.: sc-37007) oligonucleotides for the knockdown studies were purchased from Santa Cruz Biotech (Santa Cruz, CA, USA). TransFectin^TM^ Lipid Reagent (BioRad, Hercules, CA, USA) was used for transfection. Unless specified, all other materials were purchased from Sigma-Aldrich.

### 4.2. Establishment of the CRISPR/Cas9 System Targeting Deletion of the miR-371/372/373 Cluster and the miR-371/372/373 Promoter

The sequences spanning *miR-371/372/373* cluster and *the miR-371/372/373* promoter region were sent to the ATUM (https://www.atum.bio/eCommerce/cas9/input) portal in order to design small oligonucleotides that would guide the Cas9 complex and allow it to cleave the downstream sequence of the PAM (Protospacer Adjacent Motif, NGG) site. The oligunocleotides (Table S1) containing the guide sequences were annealed to form double stranded DNA and cloned into All-in-one pSpCas9-BB-2A-GFP-PX458 vector (Cat No.: 48138, Addgene, Cambridge, MA, USA) after cleavage by the *BbsI* restriction enzyme. Transfected cells expressing green fluorescence were sorted using a FACSAria cell sorter (BD Biosciences, Franklin Lakes, NJ, USA). Cell subclones derived from a single cell were achieved by the limited dilution method followed by population expansion. DNA from the expanded cells was isolated and subjected to PCR amplification to detect the potential presence of the required deletion (Table S2). In addition, the PCR product was cloned and sequenced to verify the deletion. The subclones identified as having the appropriate cluster and promoter deletions were designated the c-series and the p-series, respectively.

### 4.3. Establishment of the CRISPR-dCas9 SAM System to Allow Endogenous miR-371/372/373 Expression

The PAM sequences used for dCas9 recognition, which were localized at a position within 200 bp upstream of the TSS, were retrieved by E-crisp (http://www.e-crisp.org/E-CRISP/) *in silico* module. Oligonucleotides containing guide sequences (Table S3) to recognize the PAM were ligated into sgRNA (MS2) cloning backbone (Cat No.: 61424, Addgene) to achieve constructs that could be used for promoter activation. TransFectin Lipid Reagent (BioRad) was used for plasmid transfection. Upregulation of *miR-371/372/373* expression following transfection denoted endogenous induction.

### 4.4. Cell Viability and Apoptosis Assay

The 3-(4,5-Dimethylthiazol-2-yl)-2,5-diphenyltetrazolium bromide (MTT) assay was used to measure growth rate and drug response. The ARY009A Apoptosis Antibody Array (R&D Systems, Minneapolis, MN, USA) was used to measure the various changes in protein expression and the procedure followed the manufacturer’s instructions. Cells were harvested and stained with PI and annexin V-FITC using an appropriate kit (Cat No.: 556547, BD Biosciences, Franklin Lakes, NJ, USA) in order to measure apoptosis; this was done using a CytoFLEX flow cytometry (Beckman Coulter, Palo Alto, CA, USA).

### 4.5. Xenotransplantation

For subcutaneous tumor induction in nude mice (National Applied Research Laboratory, Taipei, Taiwan), 1 × 10^6^ cells were injected into the flank of these mice. The mice were sacrificed at the 4th week [17]. Tumor volume was calculated using the 0.5ab^2^ formula (a, greatest diameter; b, shortest diameter). All animal studies were carried out in accordance with the guidelines of the National Yang-Ming University Institutional Animal Care and Use Committee. Approval No.: 1070605, Approval Date: 11 June 2018.

### 4.6. qPCR Analysis

The expression of *miR-371/372/373* and various other mRNAs were analyzed using the TaqMan qPCR system (Table S4).

### 4.7. Western Blot Analysis

Western blot analysis followed our previous protocols [17]. The primary antibodies are listed in Table S5. The signal levels of the various tested proteins were normalized against GAPDH.

### 4.8. Statistics

Comparisons among test groups were carried out using the Mann–Whitney test and two-way analysis of variance (ANOVA). A *p* value < 0.05 was considered to be statistically significant. *, *p* < 0.05; **, *p* < 0.01; ***, *p* < 0.001; ****, *p* < 0.0001; *ns*, not significant.

## Supplementary Materials

Supplementary materials can be found at https://www.mdpi.com/1422-0067/21/24/9442/s1.

## Abbreviations

C19MC	Chromosome 19q13 miRNA cluster
CRISPR	Clustered, regularly interspaced, short palindromic repeats
dCas9	Deactivated Cas9
DKK1	Dickkopf 1
OSCC	Oral squamous cell carcinoma
MTT	3-(4:5-Dimethylthiazol-2-yl)-2,5-diphenyltetrazolium bromide
PAM	Protospacer Adjacent Motif
SAM	Synergistic activation mediator
Tcf/Lef	T-cell factor/lymphoid enhancer factor
TSS	Transcription start site
Wnt	Wingless-related integration site
