# Supplementary material for: Activation of the miR-371/372/373 miRNA Cluster Enhances Oncogenicity and Drug Resistance in Oral Carcinoma Cells
Source: Int J Mol Sci. 2020 Dec 11;21(24):9442. doi: 10.3390/ijms21249442 (PMC7764723; doi:10.3390/ijms21249442)
Supplement: Supplementary file 1 [file ijms-21-09442-s001.pdf]

Supplementary Tables

Table S1. Oligonucleotides for constructing CRISPR/Cas9 sgRNA vectors

	**Oligonucleotide**	**Sequence**
CRISPR/Cas9-1 miR-371/372/3733 cluster 5'sgRNA promoter 3'sgRNA	Sense	CACCGCAATGATACGTCCACACCTC
	Antisense	AAACGAGGTGTGGACGTATCATTGC
CRISPR/Cas9-2 miR-371/372/373 cluster 3'sgRNA	Sense	CACCGAGTTCCCGGGCTTCTCTGCG
	Antisense	AAACCGCAGAGAAGCCCGGGAACTC
CRISPR/Cas9-3 miR-371/372/373 promoter 5'sgRNA	Sense	CACCGTGAGCACGTACTCCCGCAGC
	Antisense	AAACGCTGCGGGAGTACGTGCTCAC

Table S2. Primers used for PCR reaction to detect deletion

	**Primer**	**Sequence**
miR-371/372/373 cluster deletion detection	Forward 2 (F2)	TGAGTGGATGACTGGTGGAA
	Forward 3 (F3)	CTGTGACCAAGGGGCTGTAT
	Reverse 3 (R3)	CTGTGGCATTGTCCGTGTAG
miR-371/372/373 promoter deletion detection	Forward 1 (F1)	TCATCCAGGTGGTTCACAAA
	Reverse 1 (R1)	CACACCACTGCACTCCATTC
	Reverse 2 (R2)	AGGAAGGAACACGTGTGAGG

Table S3. Oligonucleotides for constructing CRISPR/dCas9 SAM vectors

	Oligonucleotide	Sequence
CRISPR-dCas9 SAM1	Sense	CACCGTTAATCCTATCAAAGTTGAG
	Antisense	AAACCTCAACTTTGATAGGATTAAC
CRISPR-dCas9 SAM2	Sense	CACCGACCAGGGGGAATGAGAGGG
	Antisense	AAACCCCTCTCATTCCCCCTGGTC
CRISPR-dCas9 SAM3	Sense	CACCGGAGACCAGGGGGAATGAGA
	Antisense	AAACTCTCATTCCCCCTGGTCTCC
CRISPR-dCas9 SAM4	Sense	CACCGGCTTGGGGCGGAGACCAGG
	Antisense	AAACCCTGGTCTCCGCCCCAAGCC
CRISPR-dCas9 SAM5	Sense	CACCGTGGCTTGGGGCGGAGACCA
	Antisense	AAACTGGTCTCCGCCCCAAGCCAC
CRISPR-dCas9 SAM6	Sense	CACCGGTGGCTTGGGGCGGAGACC
	Antisense	AAACGGTCTCCGCCCCAAGCCACC

Table S4. TaqMan primers used in this study

Gene	Cat No
has-*miR-371*	000559
has-*miR-372*	000560
has-*miR-373*	000561
RNU6B	001093
Bad	Hs00188930_m1
Bax	Hs00180269_m1
MYADM	Hs01881097_s1
PRKCG	Hs00177010_m1
GAPDH	Hs02786624_g1

Table S5. Primary antibodies used in this study

Protein	Origin	Molecular weight (kDa)	Supplier
DKK1	Rabbit	38	Abcam
LATS2	Rabbit	120	Bethyl
p62	Mouse	62	Santa Cruz
SPOP	Rabbit	42	Proteintech
YOD1	Rabbit	38	Abcam
ZBTB7A	Hamster	72	Santa Cruz Biotechnology
AKT	Mouse	60	Santa Cruz Biotechnology
pAKT	Rabbit	60	Cell signaling
ERK	Rabbit	42, 44	Cell signaling
pERK	Rabbit	42, 44	Cell signaling
FAK	Rabbit	125	Santa Cruz Biotechnology
pFAK	Goat	125	Santa Cruz Biotechnology
Src	Mouse	60	Cell signaling
pSrc	Rabbit	60	Biosource
H-Ras	Mouse	21	Santa Cruz Biotechnology
K-Ras	Mouse	21	Santa Cruz Biotechnology
Pan-Ras	Mouse	21	Santa Cruz Biotechnology
NRF2	Rabbit	68	Sigma-Aldrich
Rho A	Mouse	24	Santa Cruz Biotechnology
Active β-catenin	Mouse	92	Merck Millipore
GAPDH	Mouse	37	Santa Cruz Biotechnology

Supplementary figures

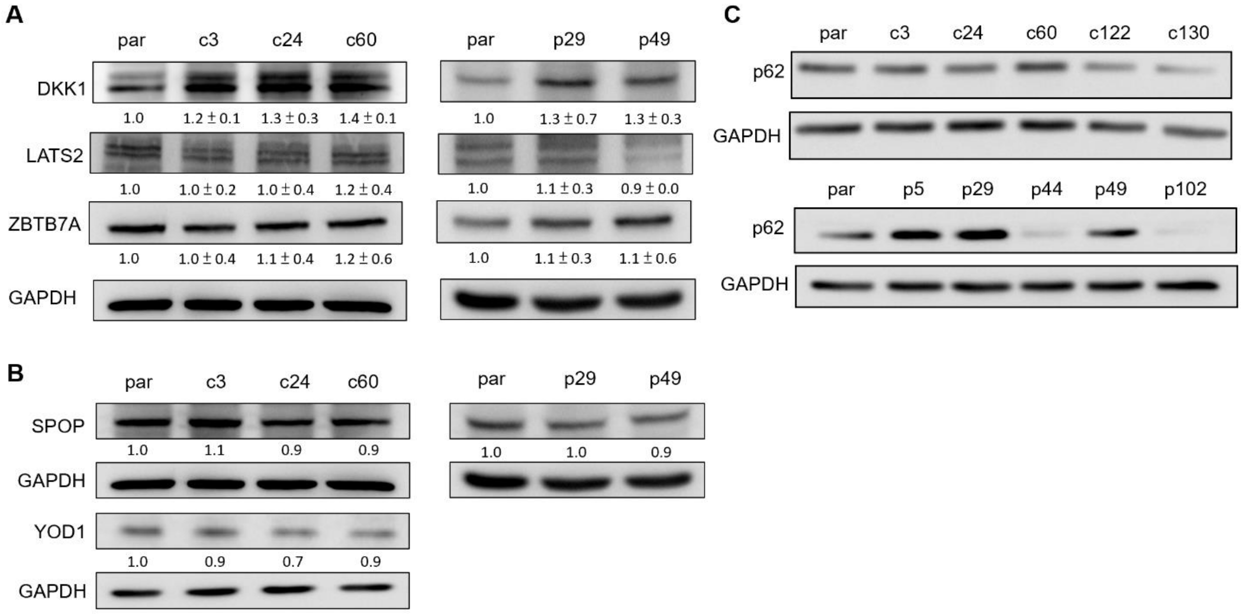

Figure S1. Western blot analysis of protein expression in parental cells and the deletion subclones. (A) DKK1, LAST2 and ZBTB7A. Data shown are means ± SE from at least triplicate analysis. (B) SPOP and YOD1. Solitary analysis. (C) p62. Solitary analysis. Since the expression pattern of p62 is irregular across the subclones, quantification was not performed. par, parental cell.

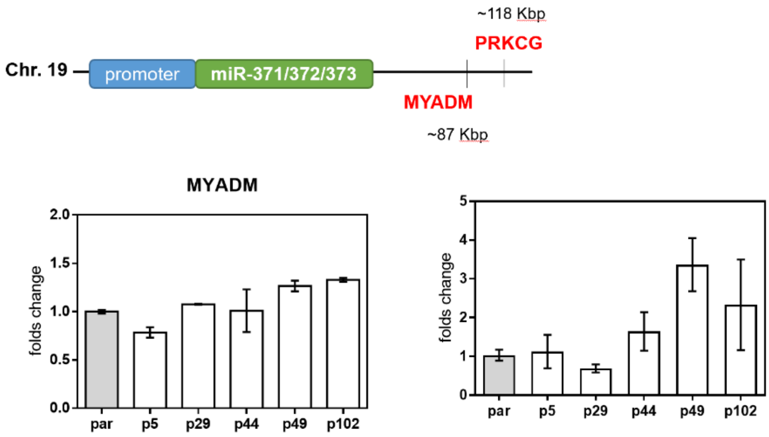

Figure S2. qPCR analysis of *MYADM* and *PRKCG* mRNA expression. Upper, diagram depicts the location of these genes relative to the *miR-371/372/373* promoter on chromosome 19. Lower, quantification of the parental cell and the *miR-371/372/373* promoter deletion subclones. par, parental cell.

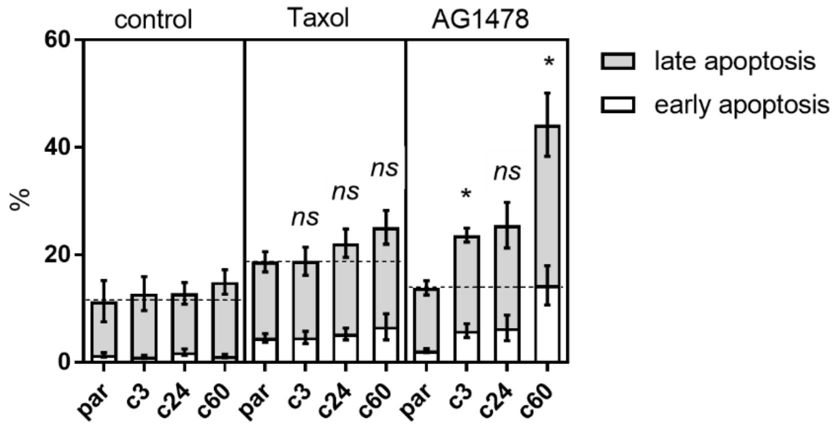

Figure S3. Quantification of the apoptosis cell fraction of the parental cells and the *miR-371/372/373* cluster deletion subclones after treatment ith either 35 nM taxol or 30 μM AG1478 for 48 h. par, parental cells.

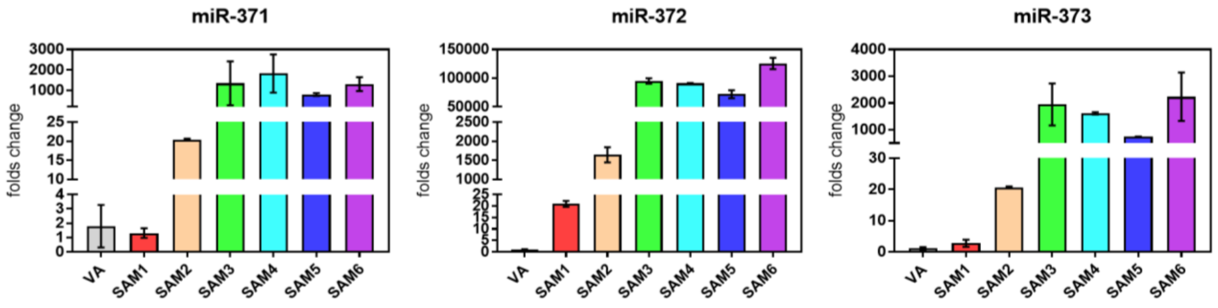

Figure S4. qPCR analysis of *miR-371/372/373* expression in cells after transfection with the SAM1 – SAM6 constructs compared to vector alone (VA) for 24 h.

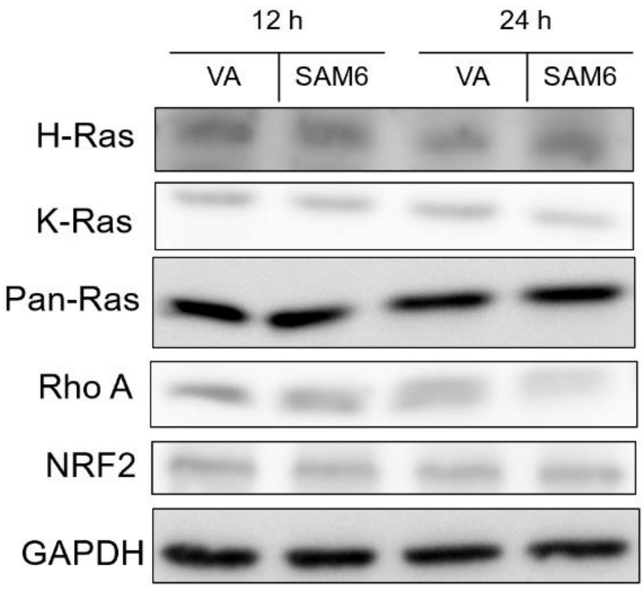

Figure S5. Representative Western blot analysis to detect the H-Ras, K-Ras, Pan-Ras, Rho A and NRF2 protein expression in cells after transfection of the SAM6 constructs or vector alone (VA) for 12 h and 24 h.
